# 

**Published:** 1846-07

**Authors:** 


### WESTERN JOURNAL

OF

### MEDICINE AND SURGERY.

### EDITORS.

---

DANIEL DRAKE, M.D.,

PROFESSOR OF PATHOLOGY AND THE PRACTICE OF MEDICINE IN THE MEDICAL INSTITUTE OF LOUISVILLE:

LUNSFORD P. YANDELL, M.D.,

PROFESSOR OF CHEMISTRY AND PHARMACY IN THE SAME:

THOMAS W. COLESCOTT, M. D.

### TO CORRESPONDENTS.

We are indebted to Drs. Fry, Harper, Affleck, and Gordon for communications, which will appear as fast as we can make room for them.

---

### BOOKS RECEIVED.

Dunglison's Medical Dictionary, sixth edition, much enlarged, Manual of the Diseases of the Teeth, by Robert Arthur, D.D.S., and a Treatise on Fevers by Tweedie and Clymer.

### RECEIPTS OF MEDICAL JOURNAL FOR JUNE, 1846.

| | |
|---|---|
| Dr. J. M. Johnston, Fredonia, Ky., | $ 2 00 |
| J. H. Bryant, Benton, Ky., | 5 00 |
| G. W. Holbrook, Waupaukonetta, Ohio, | 10 00 |
| B. A. Wheat, Neetsville, Ky., | 5 00 |
| W, Miller, Hitesville, Ill., | 5 00 |
| D. B. Rice, Oquawka, Ill., | 10 00 |
| A. G. Stevenson, Kirksville, Ohio, | 3 00 |
| J. B. Baird, South Union, Ky., | 5 00 |
| S. L Bearce, Decature, Ohio, | 3 00 |
| W. Long, New Maysville, Indiana, | 5 00 |
| W. C. Maury, Somerville, Tenn., | 5 00 |
| J. A. Green, Covington, Tenn., | 5 00 |
| M. W. Armstrong, Milton, Tenn., | 5 00 |
| Dyar, Hartsville, Tenn., | 5 00 |
| J. N. McMillin, Newark, Ohio, | 20 00 |
| J. G. Norwood, Madison, Indiana, | 5 00 |
| J. M. Bell, Dublin, Indiana, | 5 00 |
| McNally, Kingston, Ohio, | 10 00 |
| J. Richie, Franklin, Indiana, | 5 00 |
| Drs. A. & M. Dunlap, Greenfield, Ohio, | 10 00 |
| Barlow & Logan, Palestine, Illinois, | 25 00 |

